# Caring for the Adolescent

**Published:** 1921-04-09

**Authors:** 


### Caring for the Adolescent.

### AN ILLUSTRATION AND A MORAL.

A correspondent who has in the past few weeks developed an interest in the subject of industrial welfare, writes us a long letter in which he asks especially for information as to the manner in which the Industriail Welfare Society concerns itself about adolescents. Our reply must be that in the first place the State, through the Education Act of 1918, took upon itself the responsibility of caring for the adolescent—a responsibility which we are sorry to say it has now indefinitely postponed. But we cannot do better than refer our correspondent to the speech delivered by the President of the Board of Education himself at a recent London Conference on industrial welfare. On that occasion Mr. Fisher pointed out, clearly enough, that every year some 600,000 children leave the elementary schools after a careful course of education, and go out to earn their living in what is euphemistically called sometimes the University of Life. Of these 600,000 children, a few, but generally after an interval during which much that is valuable is lost, find their way into evening classes. A few become attached to some organisation, some club, some cadet corps, some society, such as the boy scouts or the girl guides, which have been formed for the purpose of advancing the welfare of our adolescent population. But the vast majority of our young people in this country, Mr. Fisher admits, are left without any guiding hand or supervision other than that fortuitously supplied by a benevolent employee

**THE PUBLIC WELL-BEING**—(*continued*).

here and there during the most critical period of their lives. "How foolish it is"—and here we give the text of the President's remarks—"to spend millions and millions of money upon the elementary education of our children, and then, just at the period of life when the mind and character are plastic, and ready to receive any impressions which may come, to throw them into the turmoil of life without further guidance. According to the figures of the census of 1911, there are in this country three million boys and three million girls between the ages of ten and eighteen. Of these some 450,000 boys are attached to some kind of club or organisation, and some 300,000 girls."

What is the moral? Mr. Fisher points it exactly. But unfortunately he is the one man who above all others must bear the responsibility for not acting upon it. The moral is that some way must be found to bridge the gap. That way seemed to have been found in the continuation school provisions of Mr. Fisher's own Act. It was an admirable and promising scheme, the details of which we have in previous issues explained to our readers. It provided not only a compulsory curriculum which logically carried on the education of the boy or girl, but an influence, an atmosphere, and common centre of interest, social and intellectual, which was altogether a new departure in our educational system. Then, at the very moment when the scheme was to be put into operation—in the case of the London County Council it was so far advanced that it could not be completely withdrawn—the official fiat went forth that "in the interests of learning" the great experiment must be side-tracked. In making this admission to his audience of social workers, Mr. Fisher sought to fall back upon the assistance of the voluntary agencies, and among them he quite properly gave a high place to the Industrial Welfare Society. But that splendid organisation, now in its infancy, cannot hope and ought not to be expected to deal adequately with the vast numbers of those boys and girls who have just left school and may or may not have entered into employment in a workshop or factory. The care of the adolescent, if it is to be systematic and complete, must be the concern of the State. That the State has acknowledged its obligations is due to the sympathy and foresight of Mr. Fisher. We must still look to Mr. Fisher to see that it fulfils them. And when he publicly appeals to business men and employers of labour to co-operate with educationists in the training and well-being of youth, the business men and the employers are justified in saying to him as the head of the educational administration in the country: "Why, having put your hand to the plough have you looked back? How can we be expected to do our part if you fail in yours?"

